# Multiplexed Adaptive RT-PCR Based on L-DNA Hybridization Monitoring for the Detection of Zika, Dengue, and Chikungunya RNA

**DOI:** 10.1038/s41598-019-47862-6

**Published:** 2019-08-06

**Authors:** Erin M. Euliano, Austin N. Hardcastle, Christia M. Victoriano, William E. Gabella, Frederick R. Haselton, Nicholas M. Adams

**Affiliations:** 1 Department of Biomedical Engineering, Vanderbilt University, Nashville, TN 37235 USA; 2 Department of Physics and Astronomy, Vanderbilt University, Nashville, TN 37235 USA; 3 Department of Chemistry, Vanderbilt University, Nashville, TN 37235 USA

**Keywords:** Assay systems, Viral infection, DNA probes

## Abstract

Reverse transcription polymerase chain reaction (RT-PCR) is the gold standard for the molecular diagnosis of many infectious diseases, including RNA viruses, but is generally limited to settings with access to trained personnel and laboratory resources. We have previously reported a fundamentally simpler thermal cycling platform called Adaptive PCR, which dynamically controls thermal cycling conditions during each cycle by optically monitoring the annealing and melting of mirror-image L-DNA surrogates of the PCR primers and targets. In this report, we integrate optically-controlled reverse transcription and single-channel monitoring of L-DNAs to develop a multiplexed Adaptive RT-PCR instrument and assay for the detection of Zika, dengue, and chikungunya virus RNA with high target specific and low limits of detection. The assay is demonstrated to detect as low as 5 copies/reaction of Zika or chikungunya RNA and 50 copies/reaction of dengue RNA. The multiplexed Adaptive RT-PCR instrument is robust and has many of the features required to implement diagnostic assays for RNA viruses in settings that lack traditional laboratory resources.

## Introduction

Mosquito-borne viruses kill more than 700,000 people per year^1^, making mosquitoes the deadliest animal in the world. The *Aedes aegypti* mosquito in particular can transmit Zika, dengue, and chikungunya viruses, among others. All three can express as fever, muscular pain, conjunctivitis, and headache, but each can lead to different long-term consequences. Dengue fever can lead to life-threatening dengue shock or dengue hemorrhagic fever, chikungunya has been associated with persistent arthritis, and Zika virus has been linked to microcephaly when an expectant mother is infected during the first trimester^2–9^. Diagnostic tools for identifying which virus has infected a patient are valuable for identifying appropriate patient management strategies and reducing the risk of adverse outcomes.

Molecular diagnostics based on reverse transcription polymerase chain reaction (RT-PCR) are considered to be the most specific and sensitive methods for detecting pathogen infections and have been used successfully for years in developed countries^10^. Serum IgM testing by enzyme-linked immunosorbent assay (ELISA) for past infection have been shown to have limited reliability for diagnosing Zika virus infection because of high antibody cross-reactivity between Zika and other flaviviruses and does not provide information about when the patient was infected^11,12^. Therefore, Zika virus infection is more commonly detected by RT-PCR of viral RNA from blood or urine samples in a clinical laboratory^6,13,14^. To date, the Food and Drug Administration (FDA) of the United States has issued Emergency Use Authorization (EUA) for 19 *in vitro* diagnostic assays to be used for Zika detection and, in concert with clinical observations and patient history, to inform patient management decisions. Of these 19 authorized assays, five are based on IgM antibody detection by ELISA and 14 are based on viral RNA detection using RT-PCR or an alternative nucleic acid amplification approach. The FDA takes into consideration a number of criteria before issuing EUA, including analytical sensitivity for FDA Zika serum standards, which is made publicly available on the FDA EUA website^15^. These tests detect Zika virus RNA in the range of 500 to 5,000 copies/mL and are valuable for informing patient management decisions in clinical laboratories; however, they lack many of the features required to be accessible in in low-resource settings, where Zika infections are of particular concern. All 14 of the molecular-based FDA EUA assays require either automated or manual RNA extraction or sample preparation, between 2–7 hours of total assay time, and in most cases, instruments that require carefully regulated ambient temperatures and regular calibrations and maintenance. Furthermore, only two of the 14 assays detect dengue and chikungunya viruses, which share similar symptoms with Zika virus. Clinics in some low-resource settings have very little laboratory support, precluding the use of these expensive and time-consuming methods. Consequently, clinical diagnoses in low-resource settings are most commonly based on symptoms, which can be unreliable and nonspecific, particularly amongst pathogens that produce similar symptoms, such as those of Zika, dengue, and chikungunya virus.

Attempts have been made to develop a PCR-based diagnostic that is suitable for settings that lack trained personnel and the resources of a clinical laboratory, but to date, commercial implementation of these devices has been limited^16–20^. Current PCR thermal cyclers depend on accurate predictions of the hybridization state of the primers and targets for the thermal cycling to be controlled properly. The accuracy of the temperature predictions is based on the precision of the thermocouple measurements that are taken outside the sample tube, the reliability of the algorithms and calibrations used to estimate the temperature inside the tube, and the concentrations of molecules in the reaction that influence the primer and target hybridization states (e.g., salts, sugars, alcohols). However, at the point-of-care in many low-resource settings, it is difficult to maintain the thermal calibrations, instrument ventilation and insulation, and sample composition because of the lack of resources for regular instrument calibrations, exposure to dust and debris, variations in ambient temperature, or samples prepared with sodium, potassium, magnesium, glycerol, ethanol, heme, or urea concentrations that fall outside the reaction tolerances. For example, one of the most promising PCR-based point-of-care diagnostic instruments, the Cepheid GeneXpert, purifies and detects tuberculosis DNA from a sputum sample with relatively high sensitivity and specificity; however, the instrument requires regular maintenance and expensive thermal calibrations^21,22^, and high failure rates have been attributed to user error, poor regulation of ambient temperatures, erratic power supplies, and dust entering the system^23^.

To address some of the challenges with PCR-based diagnostics at the point-of-care, we previously developed a more direct way to control PCR thermal cycling that is tolerant to variation in ambient temperature and inconsistencies in sample preparation. This method, called Adaptive PCR, is designed to directly monitor melting and annealing of mirror-image L-DNA analogs of PCR primers and targets to inform heating and cooling phases, rather than relying on indirect temperature sensing^24^. L-DNA, the chemical enantiomer of naturally-occurring D-DNA, has ideal properties for informing reaction conditions. First, it does not hybridize to D-DNA or interact with DNA polymerase or dNPTs, and therefore, it does not interfere with PCR. Second, it has the same physical properties as D-DNA (i.e., identical hybridization/melt response to temperature and molecules that influence hybridization, such as salts, sugars, and alcohols)^25,26^. Adaptive PCR infers the hybridization state of the PCR primers and targets in real time by optically monitoring the fluorescently-labeled L-DNA sequences, which are identical in sequence to the natural D-DNA primer and target sequences of the reaction. This differs from traditional PCR, which relies on optimization of temperature setpoints that indirectly indicate the annealing of primers or melting of amplicons during thermal cycling. Because Adaptive PCR more directly monitors the critical hybridization events during the reaction, the instrument achieves a high level of control over thermal cycling conditions without the need for thermal or optical calibrations^24,27^. The approach also has a high tolerance for variations in ambient temperature and, in combination with inhibitor-tolerant enzymes, enables amplification in complex samples without sample preparation^24,28^.

To address the needs of multiplexed RNA virus diagnostics, we have added new features to the original Adaptive PCR design. First, we developed an optically-controlled reverse transcription step. Because Adaptive PCR does not use temperature sensors to monitor the reaction, we developed a method to monitor the fluorescence signal of an L-DNA molecular beacon designed to indicate the ideal temperature for the reverse transcription hold step. Second, we modified the program to accommodate multiplexing of three targets in a single tube. In the previous Adaptive PCR iteration, two of the four available optical fluorescence channels were required to monitor target melting and primer annealing. We developed a new method to monitor L-DNA melting and annealing using a single optical channel, which made another channel available for multiplexing. Third, we developed highly sensitive and specific primers and probes for real-time, multiplexed Adaptive RT-PCR of Zika, dengue, and chikungunya virus RNA in a single tube reaction. The development and evaluation of these new Adaptive RT-PCR design features has resulted in the production of a robust platform for detecting and differentiating three RNA viruses in a single reaction tube with high specificity and low detection limit.

## Results and Discussion

### Optically-controlled reverse transcription step

**Figure 1:**
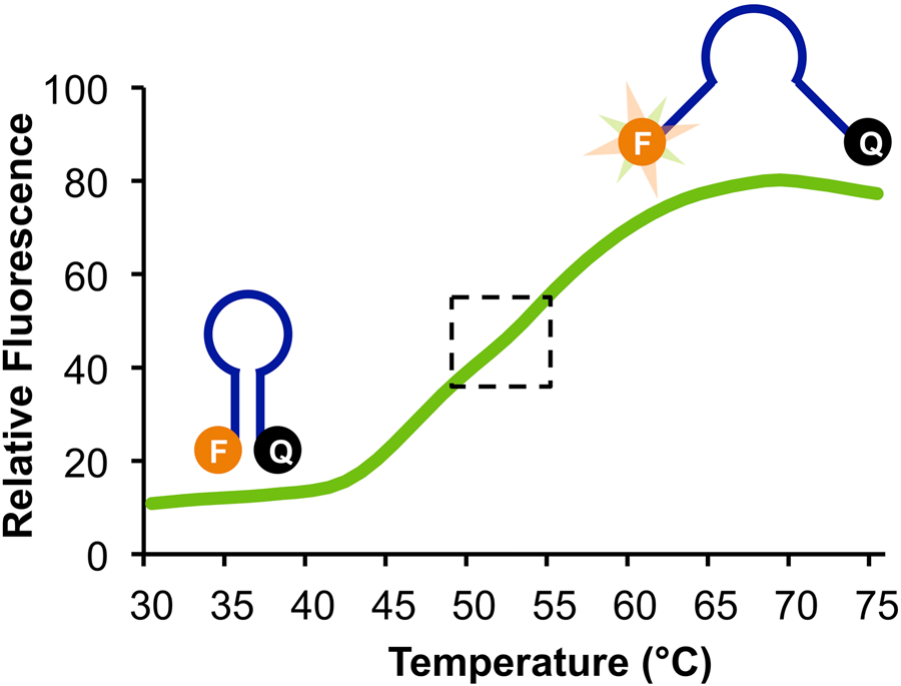
Melt curve of the L-DNA molecular beacon used to inform the reverse transcription hold step in Adaptive RT-PCR. The reverse transcription beacon was designed to have the greatest fluorescence change per degree in the temperature range most appropriate for reverse transcription (i.e., 50–55 °C), which is near the inflection point or melt temperature of the beacon (indicated by the dotted box). The Adaptive RT-PCR instrument was programmed to maintain the fluorescence at the inflection point of the melt curve. Illustrations of the reverse transcription beacon indicate the molecular conformations at the lower and higher temperatures.

To perform qPCR using an RNA target, a reverse transcription reaction is required to transcribe RNA into complementary DNA (cDNA) prior to PCR amplification. Traditionally RT-PCR requires a ~10-minute incubation step at a constant temperature, most often between 45 and 60 °C. Because the Adaptive PCR platform relies on optical sensing instead of temperature sensing to monitor the reaction steps during thermal cycling, we developed a method to optically monitor and control the reverse transcription step. To do this, a program was developed to heat the reaction to the appropriate setpoint for reverse transcription based on the optical feedback from a fluorescently-labeled temperature-sensitive molecular beacon probe (Fig. 1).

Molecular beacons are oligonucleotide sequences designed with self-complementary 5′ and 3′ ends that are labeled with a fluorophore and quencher, respectively. In the self-hybridized hairpin structure, fluorescence is quenched, and when the molecular beacons open due to some external influence such as heat, the fluorescence emission increases. Consequently, molecular beacons can be designed as effective indicators of the temperature in a solution based on the length and composition of the self-complementary stem (refer to illustrations in Fig. 1). Other research groups have taken advantage of this feature of molecular beacons to develop intracellular temperature sensors^29,30^. Our goal was to design a molecular beacon that produced the greatest change in fluorescence in the temperature range most suitable for reverse transcription, i.e. around 50 °C (Fig. 1). We chose to base the design of the stem region on the intracellular temperature sensing molecular beacon L-MB5 from Ke, *et al*.^29^, which was reported to have a melt temperature of 55 °C and a response range from 37 °C to 73 °C (Table 1). Initially, we designed the beacon with a Texas Red dye so that all three L-DNA probes (i.e., L-DNA target melt probe, L-DNA primer anneal probe, and the L-DNA reverse transcription beacon) were monitored using the same optical wavelengths. However, the fluorescence signal of the L-DNA molecular beacons and the L-DNA primer anneal probes significantly overlapped, preventing differential signal detection. The L-DNA reverse transcription molecular beacon was therefore redesigned with a FAM dye instead. While this is the same as the fluorophore conjugated to the Zika probe (see below), the consequence was that the baseline signal for the Zika amplification curve was increased relative to the other channels. This increased level of background signal had a negligible effect on the Zika amplification curve, particularly after the amplification data was normalized to the same relative scale.

**Table 1:** Oligonucleotide sequences used in these studies.

D-DNA Oligonucleotides	Zika forward primer	5′-CAGCTGGCATCATGAAGAAYC
	Zika reverse primer	5′-CACCTGTCCCATCTTTTTCTCC
	Zika hydrolysis probe	5′-**FAM**-CYGTTGTGG-**ZEN**-ATGGAATAGTGG-**IABkFQ**
	Dengue forward primer	5′-CAAAAGGAAGTCGYGCAATA
	Dengue reverse primer	5′-CTGAGTGAATTCTCTCTGCTRAAC
	Dengue hydrolysis probe	5′-**HEX**-CATGTGGYT-**ZEN**-GGGAGCRCGC-**IABkFQ**
	Chikungunya forward primer	5′-TACAGGGCTCATACCGCATC
	Chikungunya reverse primer	5′-AAAGGTGTCCAGGCTGAAGA
	Chikungunya hydrolysis probe	5′-**Cy5**-CGACCATGC-**TOA**-CGTCACAGTTAAGGA-**IAbRQSp**
L-DNA Oligonucleotides	Reverse transcription beacon	5′-**FAM**-GCGAGAAAAAAAAAAAAAAACTCGC-**BHQ1**
	Primer anneal probe	5′-**TEX**-CAGCTGGCATCATGAAGAATC
	Primer anneal compliment	5′-GATTCTTCATGATGCCAGCTG-**BHQ2**
	Target melt probe	5′-**TEX**-CTTTGTCACCGACGCCTACGTCGCAGGATCCTGGGCT GGCGGGTCGCTTCCACGATGGCCACCTCCATGGTCCTCGA
	Target melt compliment	5′-TCGAGGACCATGGAGGTGGCCATCGTGGAAGCGACCCGCC AGCCCAGGATCCTGCGACGTAGGCGTCGGTGACAAAG-**BHQ2**

LabVIEW software was programed to perform the reverse transcription hold step based on fluorescence response of the molecular beacon during heating. To avoid issues with sample-to-sample variation in the absolute fluorescence signal of the L-DNA reverse transcription beacon, the LabVIEW program plotted the derivative of the sigmoidal melt response in real time during heating, which produced a normal bell curve. The bell curve was smoothed and fit to a Gaussian distribution function, which was used to identify the peak of the derivative. This derivative peak is the point where the change in fluorescence is greatest, which is equivalent to the melt temperature of the beacon (~48 °C in the RT-PCR buffer). To hold this temperature, the LabVIEW algorithm maintained the fluorescence signal by turning the heater on when the fluorescence dropped below the value and off when the fluorescence increased above the value. This hold step continued for ten minutes. After the reverse transcription hold step, the system transitions into PCR thermal cycling, as described previously^24^.

**Figure 2:**
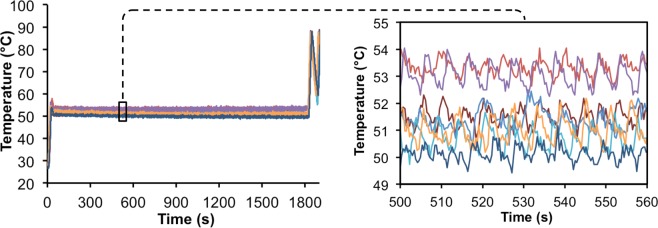
Passive temperature measurements of the reactions during 30 minute reverse transcription hold steps based on monitoring fluorescence of the L-DNA reverse transcription beacon using the Adaptive RT-PCR instrument. Measurements were collected by inserting the thermocouple directly into the reaction tube (n = 7).

To measure the accuracy of the molecular beacon-based hold step, a thermocouple was inserted into the reaction tube for seven 30-minute hold trials (Fig. 2). Although most reverse transcription kits only require 10 minutes of hold time, 30-minute trials were conducted to confirm that it was stable over longer periods of time. Sufficient thermal stability was shown in all trials, with averages of each trial ranging from 50–53.5 °C and intra-trial standard deviations of ~0.5 °C (Fig. 2, left panel). This range of temperature set points is likely the result of the lack of a definitive inflection point in the L-DNA reverse transcription beacon (refer to Fig. 1). The instrument is programed to begin the hold step after reaching the inflection point, yet because of the relatively flat melt profile (i.e., broad derivative), the inflection point is ambiguous within the range of 50–55 °C. Another explanation for this range of temperature set points is that the ESE Log optics record new fluorescence data every ~0.3 seconds, so a difference in one or two data points required to determine the “set point” can cause it to cycle around a slightly higher temperature, as time is directly related to the amount of heat delivered. This variation is within the acceptable tolerance range of most transcription reactions enzymes (i.e., 45–60 °C), however. We have confirmed that the RT-PCR performance is similar when using reverse transcription temperatures of 49, 52, or 55 °C on a Qiagen Rotor-Gene Q instrument (Supplemental Fig. 1). Another observation is that there is a periodicity of temperature fluctuations of ~0.8 °C from the average set point. As shown in the right inset of Fig. 2, the cycles occur approximately every 5–6 seconds. This periodicity is the consequence of the design of the hold step control mechanism. The instrument is programmed to switch between heating and cooling based on the absolute fluorescence value (i.e., heater on if fluorescence is above the values and heater off if fluorescence is below the values). The periodicity is directly related to the time required for the fluorescence to return to the set point after switching the heater on or off, and the time required is dependent of the ambient temperature in the room, particularly when the heater is off as cooling is passive. For example, it would require more time to cool with an ambient temperature greater than ~25 °C. Importantly, despite this periodic temperature fluctuation, the average temperatures of the seven reactions drifted on average 0.7 °C over the duration of the first 10 minutes of the hold step (i.e., the length of time of a traditional reverse transcription step) and drifted an average of 1.1 °C over the duration of the 30-minute hold step.

### Single-channel monitoring of melting and annealing

**Figure 3:**
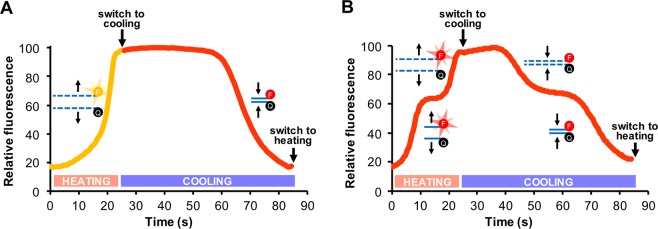
Comparison of melt and annealing curves of target and primer L-DNAs during a single thermal cycle for the two-channel (**A**) and single-channel (**B**) systems. (**A**) In the original two-channel system, the melting of HEX-labeled L-DNA targets was monitored during heating on the HEX channel (yellow curve, from 0–25 s), then the annealing of Texas Red-labeled L-DNA primers was monitored during cooling on the Texas Red channel (red curve, from 25–85 s). The original Adaptive PCR instrument was programmed to switch to cooling based on the proportion of L-DNA targets melted in the HEX channel and switch to heating based on the proportion of L-DNA primers annealed in the Texas Red channel, as indicated on the graph. (**B**) In the single-channel system, both the L-DNA target melt probes and the L-DNA primer anneal probes are labeled with Texas Red and monitored on the Texas Red channel (red curve), which results in stair-stepped fluorescence curves. The Adaptive RT-PCR instrument was programmed to switch between heating and cooling based on the second steps of the curve, which correspond to L-DNA target melting (from 15–25 s) and L-DNA primer annealing (from 55–85 s), respectively, while ignoring the first steps of the curve, which correspond to L-DNA primer melting (from 0–15 s) and L-DNA target annealing (from 25–55 s), respectively.

Multiplex PCR allows for the simultaneous detection of several different DNA targets, each with different fluorophore combinations. The Adaptive PCR device used in these studies has two Qiagen ESELog optical devices with a total of four fluorescence channels corresponding to FAM, HEX, Texas Red, and Cy5 dyes. In the earlier iteration of Adaptive PCR^24^, HEX dye was conjugated to the melt L-DNA probe and Texas Red dye to the anneal L-DNA probe (Fig. 3A). This required the use of two of the four available channels for monitoring and controlling thermal cycling. To enable the use of three channels for real-time detection of PCR amplification, a new switching program was designed to monitor L-DNA melting and annealing using a single channel. This was accomplished by labeling both the L-DNA target melt probe and L-DNA primer anneal probes with the same dye (Texas Red) and programing the device to control thermal cycling by identifying the appropriate switch point with the combined signals from the L-DNA primer and L-DNA target analogs (Fig. 3B).

The L-DNA primer anneal probe was designed to match the Zika forward primer sequence and labeled with a Texas Red dye on the 5′ end, and the L-DNA complement of this sequence was synthesized with a 3′ BHQ-2 dye (Table 1). For the L-DNA target melt probe, a G-C-rich L-DNA target melt probe from a previous Adaptive PCR assay^24^ was selected, because the melt temperature was suitable for melting the Zika, dengue, and chikungunya amplicons (i.e., ~92 °C). The L-DNA target melt probe was labeled with a 5′ Texas Red dye, and its L-DNA complement was labeled with 3′ BHQ-2 (Table 1). As in the original Adaptive PCR design, the melt point and annealing point during each cycle of the reaction are identified using the L-DNA target and L-DNA primer analogs, respectively. However, when monitoring the melting of the L-DNA primer and the melting of the L-DNA target in a single channel, they are both seen as independent sigmoidal rises in the same fluorescence channel (from 0–25 s, Fig. 3B). Therefore, the LabVIEW algorithm switches to cooling only after the L-DNA targets melt in the second rise in the fluorescence (at ~25 s in Fig. 3B). The first rise in signal during heating, which indicates primers melting from their complements, is effectively ignored. Similarly, during cooling, the annealing of the L-DNA targets and the annealing of the L-DNA primers are both seen as separate sigmoidal dips in the fluorescence signal (from 25–85 s, Fig. 3B). The program switches to heating only after the L-DNA primers anneal in the second dip in the fluorescence signal (at ~85 s in Fig. 3B). The first dip in signal during cooling, which indicates targets annealing to their complements, is effectively ignored.

It is possible to monitor the L-DNA primer anneal and L-DNA target melt probes on the same fluorescence channel, because their respective melt curves or annealing curves do not overlap during the heating and cooling phases (Fig. 3B). Initially, L-DNA target and primer annealing curves were difficult to distinguish during the cooling phase of the cycle. This seemed to be the result of the slower kinetics of annealing, which relies on diffusion for the two oligonucleotides to encounter each other and bind (as opposed to melting, which is not dependent on diffusion). The slow kinetics during cooling caused the annealing curve of the L-DNA target probe to extend and overlap with the annealing curve of the L-DNA primer probe, making it difficult to differentiate the two curves. To resolve this issue, the LabVIEW program was altered to relax the constraints for producing the first Gaussian fit.

In contrast to the variation in the temperatures that were achieved using the L-DNA reverse transcription beacon (refer to Fig. 2), the L-DNA primer anneal and L-DNA target melt probes used for PCR thermal cycling achieve highly consistent anneal and melt temperatures. This was demonstrated by completing a heating and cooling cycle from 50 to 95 to 50 °C on ten individual RT-PCR sample tubes containing the Texas Red-labeled L-DNA primer anneal and L-DNA target melt probes in RT-PCR buffer (see Supplemental Fig. 1). While there is some variation in the absolute levels of fluorescence, the melt and anneal temperatures are consistent between samples, with standard deviations ranging between 0.15 and 0.39 °C. This consistency in melt and anneal temperatures between replicates is likely the result of the distinct inflection points in the melt and anneal profiles of these probes, in contrast to the more ambiguous melt profile of the L-DNA reverse transcription beacon (refer to Fig. 1).

### Adaptive RT-PCR detection of Zika, dengue, and chikungunya RNA

**Figure 4:**
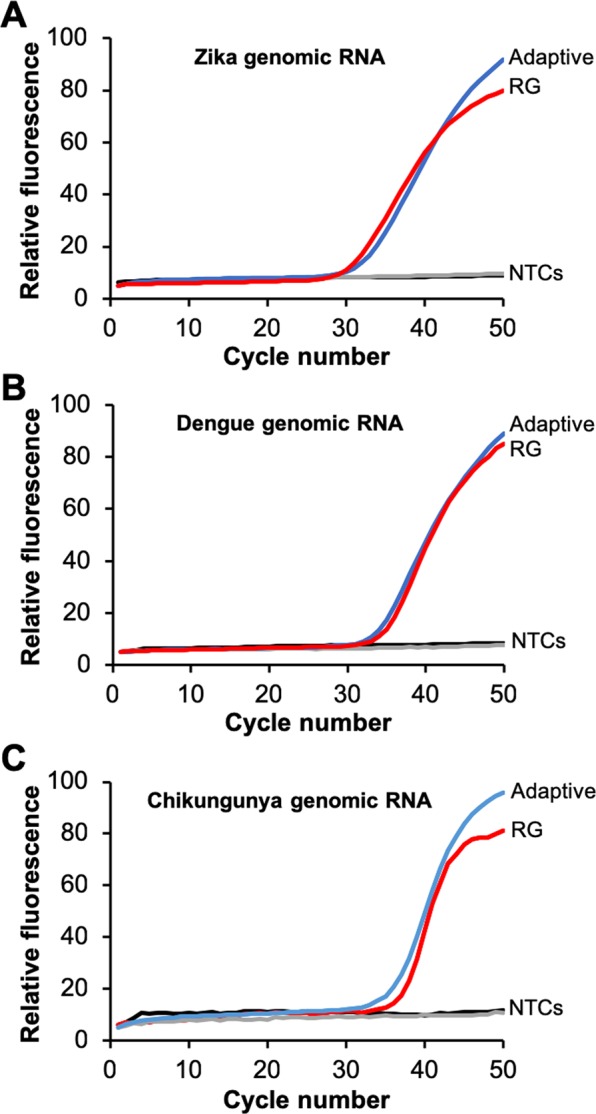
Comparison of amplification curves of 500 copies/reaction and 0 copies/reaction (NTC) of genomic viral RNA using Adaptive RT-PCR (blue) and standard RT-PCR (red) on a Qiagen RotorGene Q instrument for Zika (**A**), dengue (**B**), and chikungunya (**C**) virus. Each assay contains the same reaction mixture including all three primer and probe sets and L-DNA probes. Each amplification curve is the average of three independent runs. Individual runs are shown in Supplementary Fig. 2.

Building on this single-channel Adaptive RT-PCR design, a multiplexed RT-PCR assay was developed for Zika, dengue, and chikungunya RNA targets based on primer and hydrolysis probe sequences obtained from ATCC. The Zika forward primer was selected as the representative sequence for the L-DNA primer anneal probe in the multiplexed reaction, because the temperature at which it anneals in our system (60 °C) matches the annealing temperature used in the individual Zika, dengue, and chikungunya RT-PCR protocols. To confirm efficacy of the multiplexed assay design, Adaptive RT-PCR reactions containing the L-DNA reverse transcription beacon and anneal/melt probes plus all three PCR primer and hydrolysis probe sets were prepared. RNA from each of the three viruses was added to the reactions at 500 copies/reaction. The samples were split then RT-PCR was performed in parallel on both a Qiagen Rotor-Gene Q 5-plex HRM instrument and on the Adaptive RT-PCR instrument (Fig. 4). The amplification curves of both instruments appear similar for each virus RNA, based on the similarities in the quantification cycle (C_q_). The C_q_ differed by less than 1 cycle between the commercial Rotor-Gene Q and the Adaptive RT-PCR instrument for Zika and dengue samples, and differed by less than 2 cycles for chikungunya samples (refer to Supplemental Fig. 2). These data also confirmed that the PCR primers and probes do not amplify non-target genomic RNA, as none of the no template negative controls amplified.

**Figure 5:**
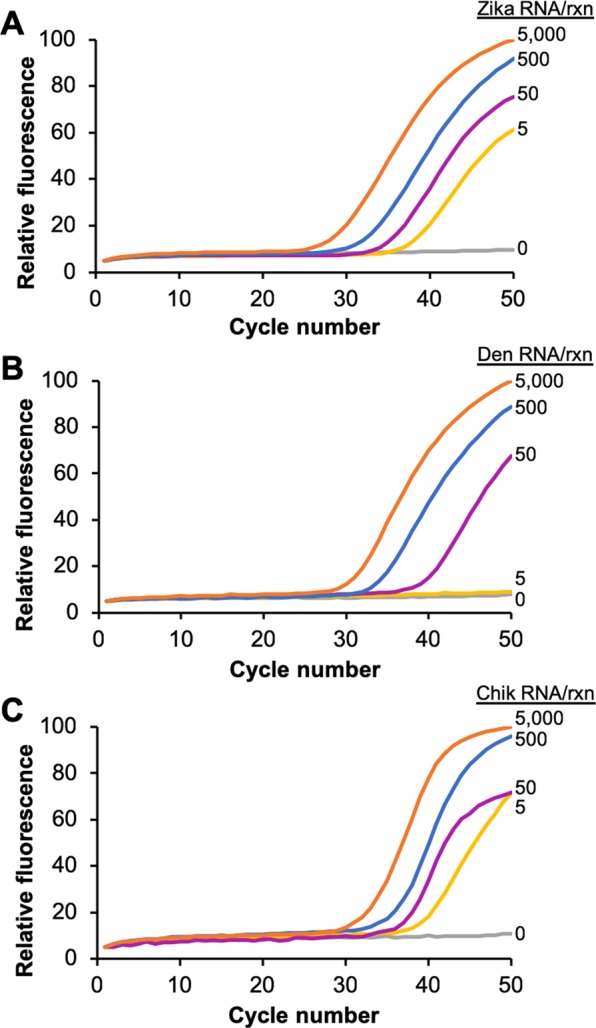
Adaptive RT-PCR of a standard dilution series of 5,000, 500, 50, 5, and 0 copies of genomic RNA from Zika (**A**), dengue (**B**), and chikungunya (**C**) virus. Each assay contains all three PCR primer and hydrolysis probe sets and L-DNA probes. Each amplification curve is the average of three independent reactions.

To determine the limit of detection of each of the assays, Adaptive RT-PCR was performed using a serial dilution of target RNA concentration for each virus (Fig. 5). Each of the concentrations of Zika virus amplified uniformly from 5,000 to 5 RNA copies/reaction, which is equivalent to 1,000 to 1 RNA copies/µL in the RNA sample (Fig. 5A). This indicates that the assay is likely sensitive enough to detect viral loads in the range of 10 copies/µL and 1 copy/µL, which have been described as necessary to detect Zika virus in blood and serum, respectively^31^. For dengue virus, the assay detected as low as 50 RNA copies/reaction, or 10 RNA copies/µL (Fig. 5B). This was not quite the limit of detection required to detect the “very low” end of the clinically-relevant viral load range, which is considered to be 1 copy/µL of dengue virus RNA^32^. The synthetic genomic RNA target used in these studies represents type-1 dengue virus. Chikungunya virus concentrations were uniformly detectable down to 5 copies/reaction, or 1 copy/µL (Fig. 5C). This limit of detection seems to be in the range of the reported average viral loads for chikungunya, which are approximately 100 RNA copies/µL of patient sample for symptomatic patients and 1 copy/µL for asymptomatic patients^8^. One explanation for why the dengue assay has a limit of detection that is an order of magnitude higher than the Zika and chikungunya assays is the greater number of mixed nucleotides present in the dengue primer and probe sequences. The dengue sequences have a total of four mixed nucleotides in positions 14 of the forward primer, 21 of the reverse primer, and 8 and 16 in the hydrolysis probe (denoted as Y or R in Table 1). The dengue primers were designed with a mixed ratio of nucleotides in these positions to ensure the detection of all four dengue virus serotypes, which have single nucleotide polymorphisms in those positions^33^. The consequence is that just 50% of the forward primers, 50% of the reverse primers, and 25% of the hydrolysis probes are identical in sequence to the dengue serotype 1. In contrast, the Zika sequences have two mixed nucleotides and the chikungunya sequences have none.

The limits of detection of the Adaptive RT-PCR Zika, dengue, and chikungunya assays are five to six orders of magnitude lower than the lowest template concentrations used in our first Adaptive PCR report^24^. Notably, the primers used in those previous studies were later determined to have self-complementarity sufficient to create hairpins during thermal cycling using primer analysis software. Despite this shortcoming, they demonstrated the concept of L-DNA-controlled Adaptive PCR well, given that the L-DNA primers used to identify the anneal step reflected the design and hybridization properties of the primers. The much lower limits of detection in these studies is likely the result of better quality primer and probe designs using in the Zika, dengue, and chikungunya assays.

### Specificity of multiplexed adaptive RT-PCR

**Figure 6:**
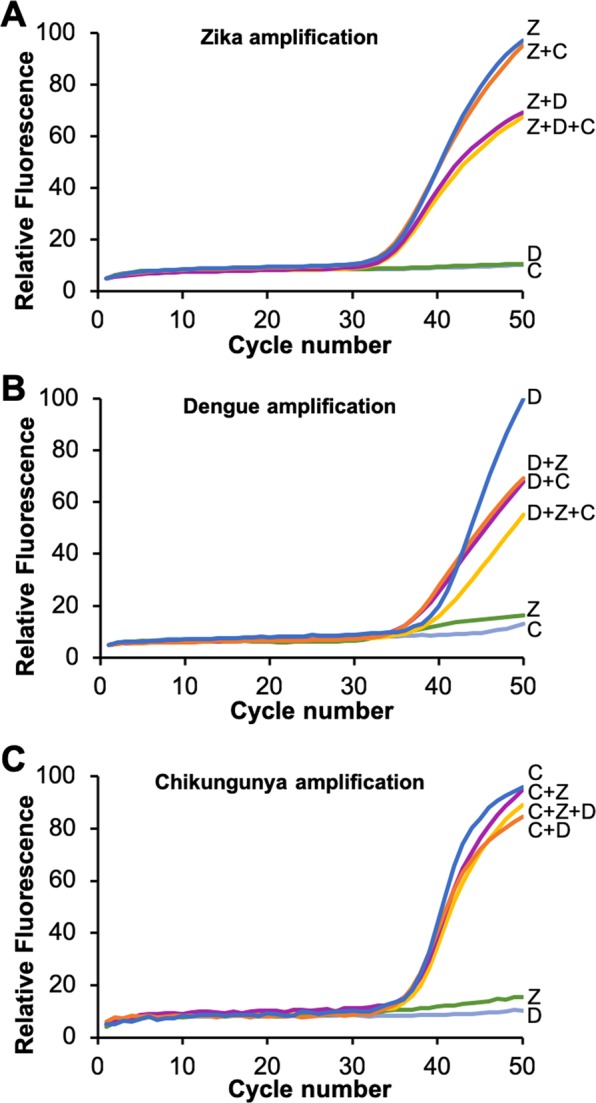
Adaptive RT-PCR amplification curves of 500 copies of Zika (**A**), dengue (**B**), and chikungunya (**C**) genomic RNA alone (blue curves), with 500 copies of genomic RNA from another virus present (magenta and orange curves), and with 500 copies of genomic RNA from both other viruses present (yellow curve). Amplification curves with non-target RNAs added (green and grey) are also included. Each assay contains the same reaction mixture including all three primer and probe sets and L-DNA probes. Each amplification curve is the average of three independent runs.

Next, the specificity of the Adaptive RT-PCR assay was evaluated by combining Zika, dengue, and chikungunya virus RNA in a single reaction. Because 50 copies/reaction was the lowest concentration consistently detectable in all three targets, that concentration was used to test detection of combinations of targets (Fig. 6). All reactions amplified within one cycle for each virus RNA in combination with each or with both of the other targets. Notably, the chikungunya assay performed nearly identically with Zika, dengue, or both Zika and dengue RNA present (Fig. 6C). In two particular combinations, some variation in the amplification curves was observed. When Zika amplified in the presence of dengue (Z + D and Z + D + C curves in Fig. 6A), the C_q_ was nearly identical, but the amplification efficiency was reduced (flatter curves). Also, when dengue amplified in the presence of Zika or chikungunya (D + Z, D + C, and D + Z + C curves in Fig. 6B), the C_q_ was approximately 2 cycles lower, but the amplification efficiency was reduced (flatter curves). One explanation for the variation is competition between the non-target RNAs or cDNAs for the primers. For example, if dengue primers bind with low affinity to some sequence of the Zika genomic RNA, that the number of primers available for dengue amplification would be reduced. Further optimization of primer and PCR hydrolysis probe design in the context of the non-target RNAs and cDNAs has the potential to improve these results; however, studies with clinical specimens would be required to determine the effect these reduced amplification efficiencies have on overall assay performance.

## Conclusions

We have developed an assay for the Adaptive RT-PCR system to detect the presence of the viral genome of Zika, dengue, and chikungunya viruses in the reaction down to 5 (Zika, chikungunya) or 50 (dengue) copies/reaction. These limits of detection are within the range of current Zika tests authorized for use by the FDA^15^, near the limits of the lowest viral loads described in clinical Zika and chikungunya specimens^8,31^, and within an order of magnitude of the range required to detect the lowest dengue viral loads^32^. Furthermore, the optics-based reverse transcription program was reproducible, and the multiplexed reaction was specific to each of the three targets. The multiplexed Adaptive RT-PCR instrument and assay for Zika, dengue, and chikungunya differentiates among viruses with similar symptoms, has many of the features required to enable point-of-care diagnosis of these RNA virus infections in low-resource settings, and can be adapted to test for other nucleic acid sequences and for other virus groups.

## Methods

### Oligonucleotide design

Quantitative Genomic RNA from Zika virus (ATCC® VR-1838DQ™), Quantitative Synthetic Chikungunya virus (CHIKV) RNA (ATCC® VR-3246SD™), and Quantitative Synthetic Dengue virus type 1 RNA (ATCC® VR-3228SD™) were purchased from the American Type Culture Collection (ATCC). Primers and PCR hydrolysis probe sequences for each were identified from previous studies and purchased from Integrated DNA Technologies (refer to Table 1). The Zika, dengue, and chikungunya primer and hydrolysis probe sequences were listed in supporting documentation to the genomic RNA standards on the ATCC website, which cites the sources of the sequences. Zika and chikungunya primers and hydrolysis probe sequences were based on an assays published and documented by the CDC^34,35^. The chikungunya primer and hydrolysis probe sequences are attributed to the Liverpool School of Tropical Medicine in the supporting documentation on the ATCC website, but a specific reference to these sequences was not identified. Hydrolysis probes were purchased from IDT as PrimeTime ZEN or TOA probes with corresponding Iowa Black quenchers, as shown in Table 1.

The L-DNA oligonucleotides used in these studies are also listed in Table 1. The L-DNA reverse transcription molecular beacon was designed based on a sequence that was reported to have a melt temperature at approximately 50 °C^29^. L-DNA primer anneal probes were designed with sequences identical to the Zika forward primer, with 5′-Texas Red dye on the primer anneal probe and 3′-Black Hole Quencher 2 (BHQ2) dye on the primer anneal complement. We used the L-DNA target melt probe and complement from a previous tuberculosis assay performed by our lab, because we already had these L-DNA targets in house and the GC-rich sequence melted at a suitable temperature for all three viruses (approximately 92 °C). All L-DNA oligonucleotides were purchased from Biomers.net GmbH (Ulm, Germany).

### Adaptive RT-PCR instrument

The Adaptive RT-PCR was developed and programmed as previously described^24^. Briefly, the device was fabricated using a forced air heater, an electronic cooling fan, and two Qiagen ESELog fluorimeters focused on a PCR sample tube. LabVIEW software running on a laptop computer was programmed to control the heater, cooling fan, and optics through National Instruments controllers and relays. The program for controlling thermal cycling was designed to monitor the derivative values of the fluorescence of the L-DNA target and primer analogs in real-time, fit the derivative to a Gaussian curve in real time, and determine the switch point between heating and cooling based on the proportion of L-DNA targets melted during heating and L-DNA primers annealed during cooling.

### Program for reverse transcription

The reverse transcription beacon was designed with a quencher and fluorophore on each end, so that as the temperature increases through the desired melt temperature of 50–55 °C, the hairpin melts apart and the fluorescence increases in a sigmoidal curve. A molecular beacon design was used instead of the complementary pairs used for monitoring target melting and primer annealing during thermal cycling, because the goal during the reverse transcription step is to incubate the reaction at the temperature most suitable for the reverse transcription enzyme, whereas the goal in thermal cycling is to identify the point at which an effective proportion of targets have melted or primers have annealed. To control the reverse transcription hold step, LabVIEW software was programmed to monitor the derivative of the fluorescence values of the L-DNA reverse transcription beacon, fit the derivative to a Gaussian curve in real time, and determine the inflection point of the raw fluorescence values. Monitoring the beacon’s fluorescence at the point where it varies most significantly with temperature enables the detection of smaller temperature fluctuations and therefore finer control of the heating element. LabVIEW was programmed to turn the heater on and off as necessary to keep the fluorescence value as close to the inflection point as possible. To do this, the program turns the heater off if the fluorescence value is greater than the set point value and turns the heater on if the fluorescence value is below the set point value. After the prescribed reverse transcription hold time, the system was programmed to transition into Adaptive PCR cycling.

To confirm the efficacy of the program, a thermocouple was inserted into the reaction to monitor temperature during a 30-minute hold step. This 30-minute temperature hold test was repeated seven times over three days to ensure consistency of the temperature range.

### Program for single fluorescence channel thermal cycling

Both the L-DNA primer anneal and L-DNA target melt probes were monitored in the same fluorescence channel using the same fluorophore label on each. Texas Red was chosen, because its quantum yield varies minimally with temperature^36^. The difference in melt temperatures between the L-DNA anneal probe and the L-DNA melt probe is approximately 25 °C. Therefore, there is a plateau of Texas Red fluorescence during heating resulting from primer anneal probes melting. Signal from target melt probes melting continues to increase after the plateau. The two curves are distinct from each other and can be distinguished by plotting the derivative and fitting it to two separate Gaussian curves. Therefore, the LabVIEW program was designed to switch from heating to cooling at two standard deviations beyond the mean of the second Gaussian curve, which corresponds to 99.7% target melting during the heating phase. During the cooling phase, the switch from cooling to heating corresponds to 99.7% primer annealing. This program of thermal cycling continued for 50 cycles.

### Program for multiplexed adaptive PCR

LabVIEW software was programmed to enable real time detection of three independent PCR products simultaneously in a single tube using three channels of the Qiagen ESELog devices corresponding to the fluorescence signal from a FAM hydrolysis probe (Zika), a HEX hydrolysis probe (dengue), and a Cy5 hydrolysis probe (chikungunya). The LabVIEW program plotted the PCR amplification data for each of these three channels in real time at the end of each cycle, immediately after switching between the cooling and heating phase.

### Adaptive RT-PCR

The SuperScript III One-Step RT-PCR kit (Invitrogen, cat no. 12574-018) was used for all reactions according to the manufacturer’s protocol. All six D-DNA primers and the three PCR hydrolysis probes were included in each reaction at 0.4 µM each (Table 1). No additional primers were included for the reverse transcription reaction, as the PCR primers doubled as reverse transcription primers in this reaction. The FAM-conjugated L-DNA molecular beacon was added at 10 nM. The L-DNA probes were each added at 50 nM and the L-DNA complements were added at 125 nM. These relative concentration ratios were selected to ensure the derivatives of fluorescence signal were approximately the same magnitude so that fluorescence signal from the L-DNA primer anneal probe and the L-DNA target melt probe would be distinct from each other.

Adaptive RT-PCR was performed using 5000, 500, 50, and 5 RNA copies/reaction for each target virus. “No Template Controls” (NTCs) with no RNA added served as negative controls. All reactions were performed in triplicate. Triplicate trials were also conducted with pairs of targets and with all three targets, each at 50 copies/reaction. Data from triplicate trials were averaged and normalized by fitting the fluorescence values from each channel to the same 100-unit scale to simplify comparisons amongst the results.

## Supplementary information

Supplementary Information

## Data Availability

The data collected during this study are available upon request.
