# Supplementary material for: Multiplexed Adaptive RT-PCR Based on L-DNA Hybridization Monitoring for the Detection of Zika, Dengue, and Chikungunya RNA
Source: Sci Rep. 2019 Aug 6;9:11372. doi: 10.1038/s41598-019-47862-6 (PMC6684530; doi:10.1038/s41598-019-47862-6)
Supplement: Supplementary file 1 — Supplementary Information [file 41598_2019_47862_MOESM1_ESM.pdf]

Supplementary Information for:
Multiplexed Adaptive RT-PCR Based on L-DNA Hybridization Monitoring for the Detection of Zika, Dengue, and Chikungunya RNA

Erin M. Euliano[1]
Austin N. Hardcastle[1]
Christia M. Victoriano[1]
William E. Gabella[2]
*Frederick R. Haselton[1,3]
*Nicholas M. Adams[1]

[1]Department of Biomedical Engineering, Vanderbilt University, Nashville, TN, 37235, USA
[2]Department of Physics and Astronomy, Vanderbilt University, Nashville, TN, 37235, USA
[3]Department of Chemistry, Vanderbilt University, Nashville, TN, 37235, USA

Supplementary Information includes:
Supplementary Figure 1
Supplementary Figure 2
Supplementary Figure 3
Supplementary Table 1

To demonstrate the consistency of the melt and anneal temperatures of the L-DNA primer anneal and L-DNA target melt probes, a heating and cooling cycle (50 to 95 to 50 °C) was completed using ten individual RT-PCR sample tubes containing the Texas Red-labeled L-DNA primer anneal and L-DNA target melt probes in RT-PCR buffer (see **Supplemental Figure 1**). While there is some variation in the absolute levels of fluorescence, the melt and anneal temperatures, as calculated by the peaks in the derivative (dF/dT), are consistent between samples.

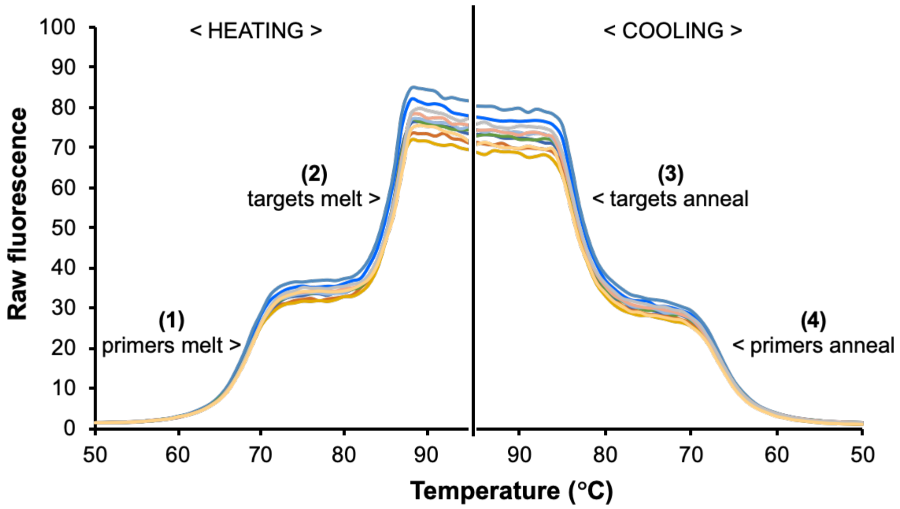

Supplemental Figure 1. The melt and anneal profile of Texas Red-labeled L-DNA primer anneal and L-DNA target melt probes in RT-PCR buffer. Each curve represents an individual RT-PCR sample tube (n = 10). Melt and anneal temperatures at each of the four inflection points were calculated using the melt analysis software as follows: 1) 68.5 ±0.15 °C, 2) 86.1 ±0.19 °C, 3) 83.2 ±0.39 °C, and 4) 66.4 ±0.36 °C**.**

To demonstrate the tolerance of reverse transcriptase for the range of temperatures measured in our system (refer to **Figure 2** in the main text), RT-PCR was performed on the Rotor-Gene Q instrument using ten-minute reverse transcription hold temperatures of 49, 52, or 55 °C. The amplification curves of the subsequent PCR were nearly identical for each of these reverse transcription temperatures (see **Supplemental Figure 2** below). These results fall in line with the product information sheet for the SuperScript III One-Step RT-PCR kit, which indicates that the reverse transcriptase enzyme "can synthesize cDNA at a temperature range of 45 - 60 °C" (https://assets.thermofisher.com/TFS-Assets/LSG/manuals/superscriptIII_onestepRTPCR_man.pdf).

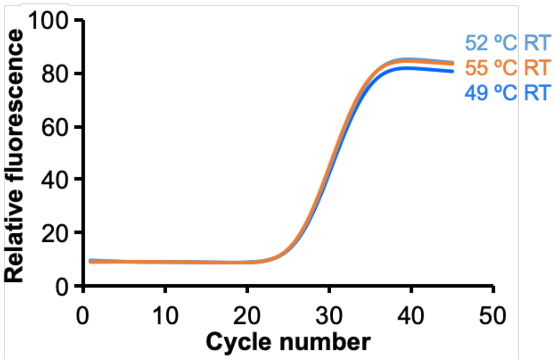

Supplemental Figure 2. Comparison of three reverse transcription temperatures performed on the Rotor-Gene Q instrument. Reactions were prepared with 5,000 copies per reaction of quantitative genomic RNA from Zika virus, as described in the Methods section of the main text. Reverse transcription was performed at 49, 52, and 55 °C in parallel on separate instruments, and then the tubes were immediately transferred into a single instrument for real-time PCR. Three tubes were tested at each temperature (nine tubes total). Each amplification curve is the average of three independent reactions.

The PCR amplification curves that were averaged in **Figure 4** of the manuscript have been plotted in **Supplementary Figure 3** below. The C_qs of the individual runs shown in **Supplementary Figure 3** were analyzed using LinRegPCR and are reported in **Supplementary Table 1** along with the averages and standard deviations. In summary, these data show that the variation between individual runs in the Rotor-Gene and the Adaptive RT-PCR instrument to be similar.

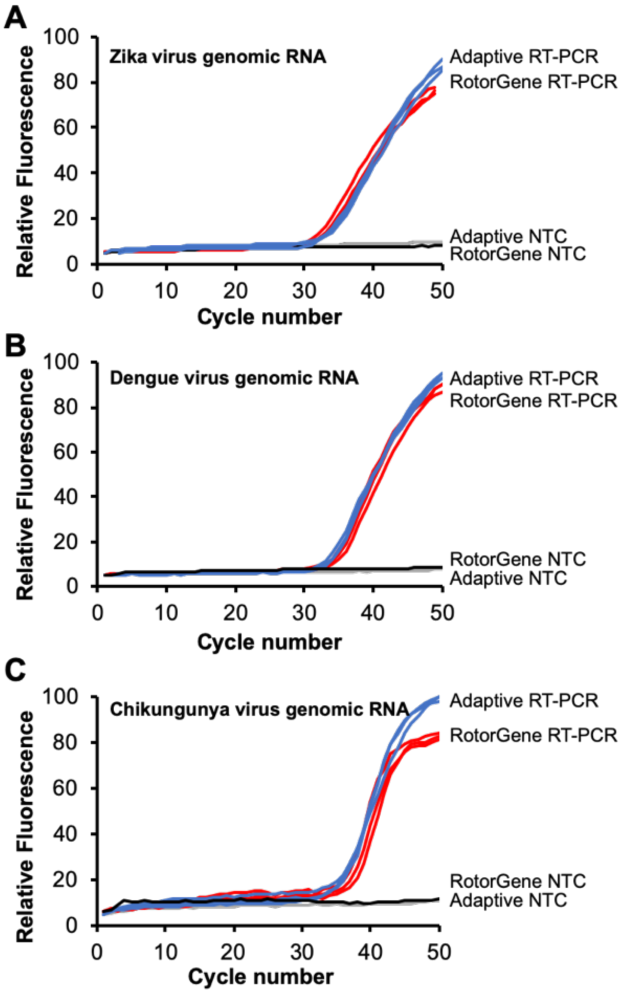

Supplemental Figure 3. Comparison of amplification curves of 500 copies/reaction and 0 copies/reaction (NTC) of genomic viral RNA using Adaptive RT-PCR (blue) and standard RT-PCR (red) on a Qiagen RotorGene Q instrument for Zika (A), dengue (B), and chikungunya (C) virus. Each assay contains the same reaction mixture including all three primer and probe sets and L-DNA probes. Each amplification curve is displayed (n = 3). Quantitative cycle (C_q), average C_q, and standard deviation values for these data are shown in **Supplementary Table 1**.

Supplemental Table 1. Quantitative cycle (C_q), average C_q, and standard deviation values for the comparison experiments performed on Adaptive RT-PCR and Qiagen Rotor-Gene Q RT-PCR shown in **Figure 4** of the main text and in **Supplemental Figure 2** of the Supplemental Information.

Target	Instrument	C_q	Average C_q	Standard deviation
Zika	Rotor-Gene Q	28.9, 28.9, 27.9	28.6	0.61
Zika	Adaptive PCR	27.4, 28.6, 27.5	27.9	0.64
Den	Rotor-Gene Q	29.7, 30.3, 30.0	30.0	0.32
Den	Adaptive PCR	28.6, 29.3, 29.3	29.1	0.40
Chik	Rotor-Gene Q	32.2, 32.9, 32.4	32.5	0.36
Chik	Adaptive PCR	30.3, 29.8, 30.5	30.2	0.33
